# Factors and Determinants for Uptake of COVID-19 Vaccine in a Medical University in Riyadh, Saudi Arabia

**DOI:** 10.7759/cureus.17768

**Published:** 2021-09-06

**Authors:** Bader A Altulaihi, Khalid G Alharbi, Talal A Alaboodi, Hamad M Alkanhal, Meshal M Alobaid, Maha A Aldraimly

**Affiliations:** 1 Family Medicine, Ministry of National Guard-Health Affairs, King Abdullah International Medical Research Center, Riyadh, SAU; 2 Family Medicine, King Saud bin Abdulaziz University for Health Sciences, Riyadh, SAU; 3 Clinical Pharmacy, King Abdulaziz Medical City, Riyadh, SAU

**Keywords:** saudi arabia, ksau-hs, covid-19, factors and determinants, coronavirus

## Abstract

Background

The coronavirus disease 2019 (COVID-19), which is also known as severe acute respiratory syndrome coronavirus-2 (SARS-CoV-2), is an infection that is caused by the novel coronavirus. COVID-19 has severely affected the public health by causing more than 200 million cases and four million deaths worldwide. There are, presently, no specific antiviral treatments for COVID-19. As immunization is one of the most successful and cost-effective health interventions to prevent this infectious disease, a number of vaccines, around 112, have been developed. In Saudi Arabia, many vaccination campaigns have already started. There are currently four approved COVID-19 vaccines but only three are available for use in Saudi Arabia.

Methods

This was a cross-sectional study in which a web-based survey was distributed to medical students in their clinical years at the College of Medicine in King Saud bin Abdulaziz University, Riyadh, Saudi Arabia. A total of 209 questionnaires were distributed. The survey assessed the demographic data, perception towards COVID-19 vaccine, barriers and predictors for accepting COVID-19 vaccine.

Results

Two-hundred and four respondents completed the survey with a response rate of 96.7%. Overall, 118 of the participants were males and 86 were females. Sixty-six percent of our participants had taken the vaccine. Of those, males and females were distributed equally in half. Fifty-three percent of the participants who had taken the vaccine aged 21-23. This age group had a significant effect on acceptance of the vaccine. Most common deterrent to taking COVID-19 vaccine was safety issues despite not having a statistical significance. In terms of motivators, the majority thought that fear of getting COVID-19 infection was the most important motivator to taking COVID-19 vaccine, which was statistically significant as well.

Conclusion

In the setting of spreading COVID-19 infection, the vaccine is still the solution to halting infection spread. Based on our findings, we see that there was a high acceptance rate (66.2%) of COVID-19 vaccine.

## Introduction

The coronavirus disease-2019 (COVID-19), which is also known as severe acute respiratory syndrome coronavirus-2 (SARS-CoV-2), is an infection that is caused by the novel coronavirus-2019 [1]. COVID-19 made a severe impact on public health by reaching more than 200 million cases and 4 million deaths worldwide [2]. Vaccines are presently the most effective method of halting the spread of COVID-19 since there are no proven medicines that are effective against COVID-19 [3]. Therefore, around 112 vaccines had been manufactured and were authorized for use in every country worldwide [4]. In Saudi Arabia, many vaccination campaigns have already started. The available vaccines in the Kingdom are Oxford- AstraZeneca, Pfizer-BioNTech and Moderna [5,6].

Healthcare workers have different perceptions on the COVID-19 pandemic. Bhagavathula, et al. [7] reported that 61% of the included healthcare workers had used social media to get information on COVID-19. A significant proportion of their participants had poor knowledge of COVID-19 transmission (n=276, 61.0%) and symptom onset (n=288, 63.6%). Another study was conducted on medical students in Iran [8] in 2020 to measure their perception of COVID-19. The authors reported that the average rate of participants practicing preventive behaviors was 94.47% and 79.60% had high level of knowledge related to COVID-19 transmission and symptom onset.

It is also important to know populations' perceptions about vaccine safety as this might be a deterrent to taking the COVID-19 vaccine. Wang, et al. [9] reported that, in August of 2020, 91% of the participants would accept the vaccine when available. Of those, 52% stated that they would get vaccinated as soon as possible, while the others intended to delay getting vaccinated until its safety was confirmed. A study was done in Saudi Arabia [10], which showed that 65% were willing to take the COVID-19 vaccine when available.

Another local study conducted in Jeddah, Saudi Arabia [11] aimed to explore the rate and factors related to acceptance of the COVID-19 vaccine. Similar to the previous study, half of the participants stated that they would accept taking the COVID-19 vaccine. Furthermore, participants were more likely to take the vaccine if they lived in the southern region of Saudi Arabia, had already taken the flu vaccine, had high levels of concern about getting COVID-19 infection, or believed that the COVID-19 vaccine should be mandatory. Also, the authors observed that the participants who had a history of vaccine refusal were less likely to take the COVID-19 vaccination.

To our knowledge, there were no studies done to explore the perception of the aspiring physicians towards the COVID-19 vaccine in Riyadh, Saudi Arabia. Therefore, this study focused on exploring the perception and knowledge of medical students at KSAU-HS towards COVID-19 vaccinations in Riyadh, Saudi Arabia.

## Materials and methods

This was a cross-sectional study that included 209 medical students in King Saud bin Abdulaziz University for Health Sciences (KSAU-HS), Riyadh Saudi Arabia. This study was approved by the International Review Boards of King Abdullah International Medical Research Center (KAIMRC), Ministry of National Guard- Health Affairs, Riyadh, Kingdom of Saudi Arabia (approval number NRC21R/133/04). The students in the university were in different batches. A batch is a group of students who are in the same year in medical school. Each group of students who enroll in medical school each year are assigned a batch number. The inclusion criteria were all medical students in KSAU-HS from batch 14, who were in the 6th year of medical school, to batch 18, who were in the 2nd year of medical school. No exclusion criteria were considered. The study's population was estimated to be approximately 784 participants, which was the total number of medical students in the college of medicine of KSAU-HS. Our sample size was estimated to be 211. This was calculated using a confidence interval of 95%, margin of error of 5%, and a response distribution of 75%. The sampling technique used in this study was a convenient sampling technique in which all eligible participants were included. The study was conducted using an online questionnaire that was adopted from similar studies conducted in Iran and Egypt which are cited below as reference 8 and 16, respectively. The questionnaire was distributed to the participants through their university e-mails. Double response was prevented by allowing participants to have one response only. This was checked by having the participants log into their e-mail accounts to confirm that they had not answered the questionnaire previously. The questionnaire was distributed to the participants in English language as all participants were medical students. The questionnaire was piloted by distributing it to 30 participants, of which 25 responses were received. It was then distributed formally to 211 participants. Data collected in the questionnaire included demographic data, perceptions, awareness, concerns and barriers regarding COVID-19 vaccine. Level of significance was set at a p-value of <0.05, and data analysis was performed using the Statistical Package for Social Science (SPSS) version 24.0.

## Results

Two hundred and four respondents completed the survey with a response rate of 96.7%. Table 1 shows the number and percentages of the participants’ demographic data. Overall, 118 of the participants were males (57.8%), and 86 were females (42.2%). Participants' ages ranged from 19 to 29 years of age. An even distribution of participants across all batches (14th-18th) was sought during data collection as evident in Table 1.

**Table 1:** Demographic data. N = number; % = percentage.

	N	%
Gender		
Male	118	57.8
Female	86	42.2
Age		
19 years	24	11.8
20 years	35	17.2
21 years	35	17.2
22 years	37	18.1
23 years	36	17.6
24 years	18	8.8
25-29 years	19	9.3
Batch		
18^TH^ (second year of medical school)	38	18.6
17^TH ^(third year of medical school)	35	17.2
16^TH ^(fourth year of medical school)	44	21.6
15^TH ^(fifth year of medical school)	42	20.6
14^TH ^(sixth year of medical school)	45	22.1

**Figure 1:**
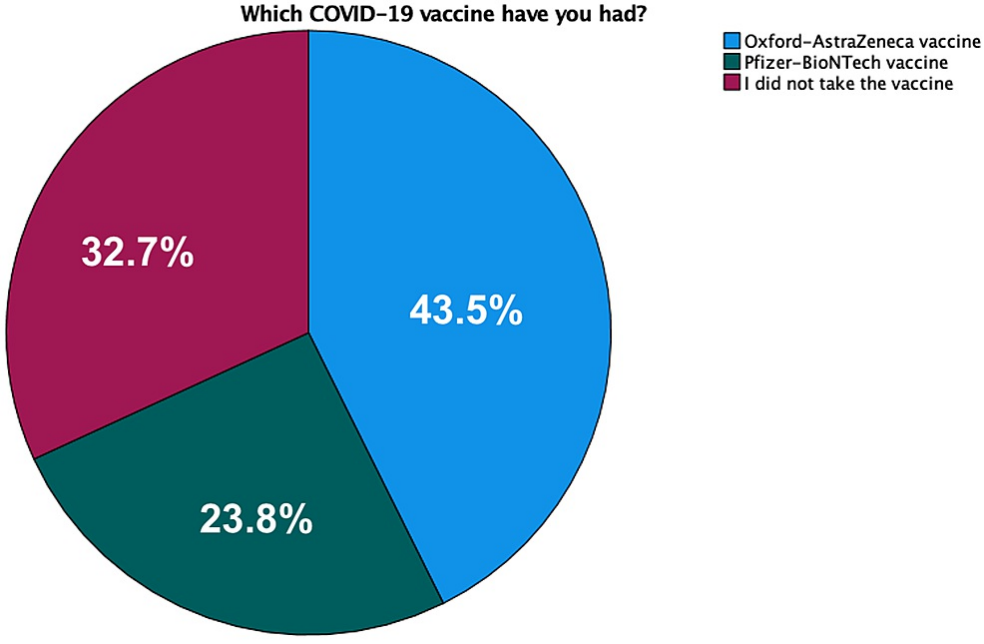
Distribution of COVID-19 vaccine brand among participants. COVID-19 = coronavirus disease of 2019.

Figure 1 illustrates the distribution of those who had taken the vaccine by brand. Of those, males constituted 55.6% while females comprised the remaining 44.4%. Half the participants (53%) who had already taken the vaccine were in the age groups of 21-23 years of age. On the other hand, half (59.4%) of those who had not taken the COVID-19 vaccine were in a slightly younger age group (19-20 years of age). Using Kruskal-Wallis test, the age group of 21-23 had a statistically significant effect on whether or not to take the COVID-19 vaccine (p-value = 0.002). Those aged 21-23 were more likely to take the vaccine compared to those younger or older. Gender, however, did not prove to have a significant effect on our participants’ decision to take the vaccine (p-value = 0.124).

Nearly all participants (99%) were aware that they were eligible for COVID-19 vaccination. Similarly, 93% of the included medical students were aware of the correct number of vaccine doses required to be immune. However, only 54% of them were aware of the number of COVID-19 vaccines available in the Kingdom of Saudi Arabia. Despite that, it was clear that our participants had an acceptable overall awareness about COVID-19 vaccines.

Thirty-one of the study’s participants (15.2%) contracted COVID-19 infection previously. Using Chi-square test, we found that contracting COVID-19 infection previously significantly affected the willingness to take the vaccine (p-value = 0.023). Those who contracted COVID-19 infection previously were more likely to take the vaccine. On the other hand, having taken the seasonal flu vaccine did not statistically affect the decision to take the COVID-19 vaccine (p-value = 0.401).

**Figure 2:**
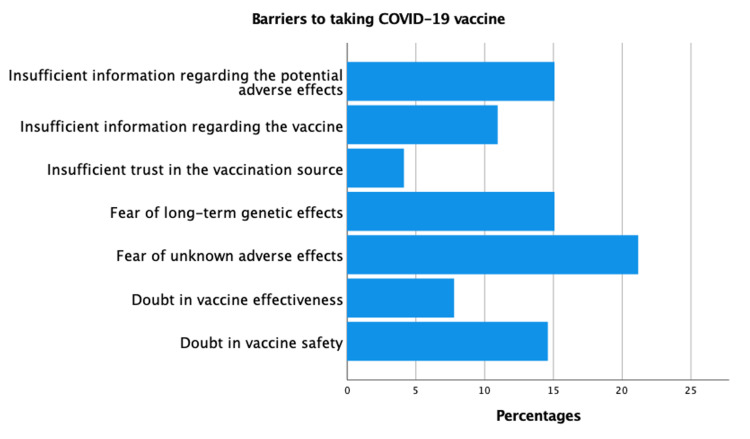
Barriers to taking COVID-19 vaccine. COVID-19 = coronavirus disease of 2019.

We asked our participants about the barriers to taking the COVID-19 vaccine. Figure 2 demonstrates the reported barriers. The most commonly reported barrier was fear of unknown adverse effects from the vaccine, followed by fear of genetic effects. The latter is especially interesting since it has not been an established side effect of the vaccine. Among the most common barriers was doubt in vaccine safety and effectiveness. Even though it is common, we were not able to find a statistical significance of this factor on the acceptance to taking the COVID-19 vaccine (p-value = 0.219).

Safety was a concern among the public before the start of this study. This was evident in our study as one-third of the medical students were ambiguous as to whether or not the vaccines had been tested rigorously enough prior to launch. The other two-thirds were distributed equally between agreement and disagreement to the previous statement. We further asked our participants about other concerns towards the COVID-19 vaccine. Seventy-nine percent of them were concerned that they might get COVID-19 infection from the vaccine. This proved to be a significant factor in deterring the participants from taking the vaccine (p-value = 0.034). 

**Figure 3:**
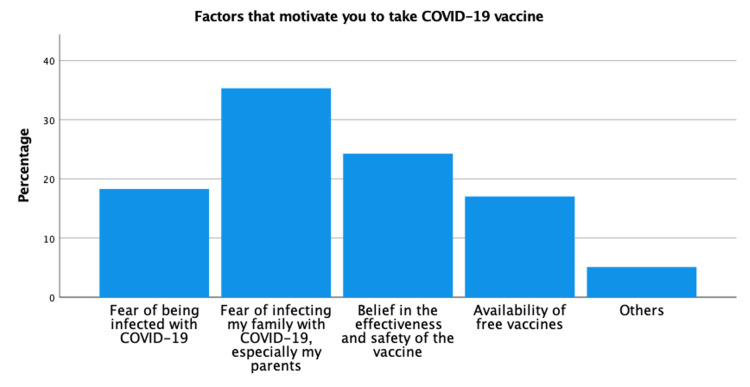
Motivators to taking COVID-19 vaccine. COVID-19 = coronavirus disease of 2019.

Many factors were stated by the study’s participants. Figure 3 shows that the most commonly cited factor was fear of infecting their family members, especially their parents. Fear of getting COVID-19 infection was also a commonly reported factor. Those who were afraid of getting the infection themselves, or transmitting it to their parents had a statistically higher likelihood of taking the COVID-19 vaccine (p-value = 0.019 and 0.034, respectively). 

## Discussion

Our study showed that the overall vaccine acceptance rate among medical students was 66.2%. This finding is in accordance with the vaccine acceptance rate reported in a study done in the United States [12]. However, a study conducted in Uganda [13] showed that only one-third of the medical students were willing to take the COVID-19 vaccine, which could be attributed to the low infection rate in Uganda [14] or cultural differences compared to Middle Eastern and Western countries.

In our study, we found that the number of medical students who had taken COVID-19 vaccination was not the same across gender. The total number of vaccinated male students was higher than female medical students. Similarly, a study conducted in Egypt [15] showed that the percentage of male students willing to take the COVID-19 vaccine was higher than their female counterparts. However, a cross-sectional study done in India [16] among medical students showed that the number of female students accepting COVID-19 vaccination was higher than those hesitant. Our finding could be attributed to the growing fears among Saudi females regarding the effect of certain COVID-19 vaccines on pregnancy and fertility since it has been wrongly assumed that COVID-19 is associated with this adverse effect. The latter, however, had been negated by the Ministry of Health in Saudi Arabia [17].

In our study, participants’ ages ranged from 19 to 29 years of age. The majority of those who had taken the COVID-19 vaccine were between the ages of 21 to 23. Statistical analyses were done on the significance of this finding which showed that the age group of 21 to 23 had a statistically significant effect on the acceptance of the COVID-19 vaccine among medical students (p-value = 0.002). Participants aged 21-23 were more likely to take the COVID-19 vaccine than other age groups. This is similar to the Egyptian study [15] which reported that the COVID-19 vaccine acceptance rate was higher among graduate students, who are usually aged 22-24, than other age groups. This finding could be explained by different factors including a higher level of education among graduates, different beliefs, or more awareness.

It is generally accepted that COVID-19 can lead to higher mortality rates or serious disease course among those with chronic diseases. Similarly, the elderly population might be more prone to serious COVID-19 infection course [18]. Therefore, people worry more about their parents getting the infection especially if they were the source of the infection. We found in this study that our participants feared transmitting the infection to a family member. This factor proved to have a statistically significant effect on our participants’ willingness to take the COVID-19 vaccine (p-value = 0.019). Similarly, the previous study conducted among medical students in Uganda [13] showed that the most commonly reported reason for acceptance of the vaccination was that students wanted to protect themselves and their relatives from COVID-19.

In the present study, we found that fear of being infected with COVID-19 had statistically increased participants' willingness to take the vaccine (p-value = 0.034). Similarly, the study conducted in Egypt [15] among medical students found that the most cited reason for getting vaccinated was the fear of being infected or affecting others.

Vaccines are one of the most important and highly effective preventive measures. Numerous infections can be prevented by vaccination and that includes COVID-19 and Influenza infection [19]. People around the world are becoming more aware of the importance of vaccination. However, we found that having taken the previous seasonal flu among the students did not affect the participants' willingness to take the COVID-19 vaccine (p-value = 0.401). In contrast to our finding, Pastorino, et al. [20] reported that Italian medical students were more likely to accept the COVID-19 vaccine if they had had the flu vaccine earlier.

There were different barriers to taking the COVID-19 vaccine as reported by our participants. One of the major reasons was the effectiveness and safety of the vaccine. Despite being commonly reported, we were not able to find any statistical significance of vaccine safety and effectiveness on the willingness to take the COVID-19 vaccine. However, the study done on students in Uganda [13] showed that students' beliefs of vaccine effectiveness were significantly associated with COVID-19 vaccine acceptability.

Despite our findings, the study has its limitations. The current study included only medical students from the college of medicine in King Saud bin Abdulaziz University for Health Sciences (KSAU-HS). Therefore, our findings may not be generalizable to represent the overall perception and attitude of medical students across the Kingdom of Saudi Arabia. This study could be improved by increasing the sample size and including medical students from different universities in different cities/regions of Saudi Arabia. The barriers and motivators for taking the COVID-19 vaccine were set earlier. Even though an “Others” option was added, very few of the participants chose to write their thoughts specifically. This is a limitation as it could narrow the respondents' thoughts when answering the question rather than starting with an open-ended question to generate more thoughts from our participants.

## Conclusions

In the setting of spreading COVID-19, the vaccine is still a breakthrough solution to controlling the infection spread. Most of the medical students took the vaccine and were willing to motivate their colleagues to do the same. Based on our findings, we see that there is an overall good perception among medical students towards the COVID-19 vaccine. This is a promising finding since our participants will, hopefully, be the future of Saudi Arabia’s health care providers. We have found that age groups of 21-23 were more likely than others to accept the vaccine. This is a finding that merits further research to explore the reasons why older individuals might be less likely to accept the vaccine.

## References

[1] Seyed Hosseini E, Riahi Kashani N, Nikzad H, Azadbakht J, Hassani Bafrani H, Haddad Kashani H (2020). The novel coronavirus Disease-2019 (COVID-19): Mechanism of action, detection and recent therapeutic strategies. Virology.

[2] (2021). WHO coronavirus (COVID-19) dashboard. https://covid19.who.int.

[3] Yadav T, Srivastava N, Mishra G, Dhama K, Kumar S, Puri B, Saxena SK (2020). Recombinant vaccines for COVID-19. Hum Vaccin Immunother.

[4] (2021). COVID-19 vaccine tracker and landscape. https://www.who.int/publications/m/item/draft-landscape-of-covid-19-candidate-vaccines.

[5] (2021). Saudi food & drug authority allows the import and use of AstraZeneca COVID-19 vaccine. https://www.sfda.gov.sa/en/news/79059.

[6] (2021). SFDA approves registration of Pfizer-BioNTech COVID-19 vaccine. https://www.spa.gov.sa/viewfullstory.php..

[7] Bhagavathula AS, Aldhaleei WA, Rahmani J, Mahabadi MA, Bandari DK (2020). Knowledge and perceptions of COVID-19 among health care workers: cross-sectional study. JMIR Public Health Surveill.

[8] Taghrir MH, Borazjani R, Shiraly R (2020). COVID-19 and Iranian medical students; a survey on their related-knowledge, preventive behaviors and risk perception. Arch Iran Med.

[9] Wang J, Jing R, Lai X, Zhang H, Lyu Y, Knoll MD, Fang H (2020). Acceptance of COVID-19 vaccination during the COVID-19 pandemic in China. Vaccines.

[10] Al-Mohaithef M, Padhi BK (2020). Determinants of COVID-19 vaccine acceptance in Saudi Arabia: a web-based national survey. J Multidiscip Healthc.

[11] Alfageeh EI, Alshareef N, Angawi K, Alhazmi F, Chirwa GC (2021). Acceptability of a COVID-19 vaccine among the Saudi population. Vaccines.

[12] Lucia VC, Kelekar A, Afonso NM (2020). COVID-19 vaccine hesitancy among medical students. J Public Health.

[13] Kanyike AM, Olum R, Kajjimu J (2021). Acceptance of the coronavirus disease-2019 vaccine among medical students in Uganda. Trop Med Health.

[14] (2021). Uganda COVID: 45,231 cases and 361 deaths. https://www.worldometers.info/coronavirus/country/uganda/..

[15] Saied SM, Saied EM, Kabbash IA, Abdo SA (2021). Vaccine hesitancy: beliefs and barriers associated with COVID-19 vaccination among Egyptian medical students. J Med Virol.

[16] Jain J, Saurabh S, Kumar P (2021). COVID-19 vaccine hesitancy among medical students in India. Epidemiol Infect.

[17] (2021). No deaths resulted from COVID-19 vaccine, MOH says. https://www.moh.gov.sa/en/Ministry/MediaCenter/News/Pages/News-2021-03-21-007.aspx.

[18] Yanez ND, Weiss NS, Romand JA, Treggiari MM (2020). COVID-19 mortality risk for older men and women. BMC Public Health.

[19] (2021). Key facts about seasonal flu vaccine. https://www.cdc.gov/flu/prevent/keyfacts.htm.

[20] Pastorino R, Villani L, Mariani M, Ricciardi W, Graffigna G, Boccia S (2021). Impact of COVID-19 pandemic on flu and COVID-19 vaccination intentions among university students. Vaccines.

